# Critical Thinking in Biology Education: Insights from Kuhn’s Paradigm Shifts

**DOI:** 10.3390/bs16020296

**Published:** 2026-02-19

**Authors:** Chao Chen, Huangdong Ma, Wencheng Liu, Guian Li, Jiyu Yang

**Affiliations:** 1 Faculty of Education, Shaanxi Normal University, Xi’an 710062, China; chenchao0527@snnu.edu.cn; 2 School of Teacher Development, Shaanxi Normal University, Xi’an 710062, China; mahuangdong@snnu.edu.cn; 3 School of Physics and Information Technology, Shaanxi Normal University, Xi’an 710119, China; 4 Biomedical Analysis Center, College of Basic Medicine, Third Military Medical University (Army Medical University), Chongqing 400038, China; yangjiyu@tmmu.edu.cn

**Keywords:** critical thinking, science education, Thomas Kuhn, paradigm shifts

## Abstract

Critical thinking (CT) is widely recognized as a central goal of science education, yet its mechanisms within specific disciplinary contexts remain underexplored. This study developed a biology-specific theoretical model of CT through qualitative analysis of high school students’ engagement with contradictory evidence. Data included pen-and-paper responses from 196 students and eight classroom dialogue transcripts, analyzed using Corbin and Strauss’s coding procedures, with sequential batching and external validation. Selective coding identified questioning—transforming multiple criteria—as the core category, supported by four major categories: evolving evaluative criteria, various types of reasoning, analysis without judgment, and the application of empirical knowledge across criteria. This model explains how learners shift between confirmation, falsification, and reconstruction when anomalies disrupt initial assumptions. To extend its theoretical reach, the model was placed in heuristic dialogue with Kuhn’s structure of scientific revolutions. The comparison highlights the cyclical nature of CT development: anomalies destabilize prevailing frameworks and trigger reorganization of evaluative criteria, fostering cognitive growth. By explicating how students engage with contradictory evidence and transform evaluative criteria, this study elucidates the emergence of critical thinking in disciplinary practice. The findings also inform the design of biology learning environments that deliberately incorporate anomalies and cognitive conflicts, and justify the integration of history and philosophy of science (HPS) perspectives to support students’ questioning, analysis, and criteria revision in authentic scientific contexts.

## 1. Introduction

Critical thinking (CT) is widely regarded as an essential skill for students in the twenty-first century and one of the most frequently discussed concepts in contemporary education and reform agendas (Cargas et al., 2017; T. J. Moore, 2011; Tian et al., 2025). It is considered vital both for personal success in academic and professional contexts and for addressing complex real-world problems through reasoning and evaluation (Hu & Bi, 2025; Tiruneh et al., 2017). Educational authorities worldwide have increasingly recognized CT as a central learning outcome (Hart et al., 2021). For example, in the United States, the Next Generation Science Standards (NGSS) highlight that preparing high-school graduates for college and careers requires not only disciplinary knowledge but also the ability to engage in evidence-based reasoning and problem solving (García-Carmona, 2025; Lead States, 2013). Employers also stress the importance of graduates who demonstrate inquiry-driven problem-solving and critical reasoning abilities, rather than relying solely on specialized technical expertise (Davies, 2013). Similarly, the most recent Chinese science curriculum standards place CT at the core of science education, emphasizing argumentation, inquiry, and evaluation as indispensable components of students’ scientific literacy (Ministry of Education of the People’s Republic of China, 2018; Tian et al., 2025). Scholars have further argued that CT enables students to distinguish between science and pseudoscience, to assess the credibility of evidence and information sources, and to form independent and well-reasoned judgments (Moura et al., 2023; Sparks & Rapp, 2011). In an age of information abundance, where unreliable content on platforms such as TikTok or Douyin can shape public attitudes, the ability to critically evaluate claims is more necessary than ever (Liu et al., 2025b). Consequently, fostering CT has become a key objective of education systems worldwide.

Despite the widespread recognition of its importance, the definition of CT remains debated (Cuypers, 2004; T. Moore, 2013; Tian et al., 2025). These ongoing conceptual disagreements have important implications for educational practice, contributing to considerable variation in assessment frameworks and uncertainty about what existing instruments actually measure (Tiruneh et al., 2017), as well as complicating decisions about how CT should be taught and fostered in classrooms. In science education, scholars and practitioners widely acknowledge the value of fostering CT (Hu & Bi, 2025), yet empirical research into how CT should be cultivated within subject-specific contexts—particularly in biology—remains limited (Mkimbili, 2024).

Thomas Kuhn’s landmark The Structure of Scientific Revolutions strongly influenced understandings of how scientific knowledge develops (Kuhn, 1962). His cyclical model of normal science, anomaly, crisis, and revolution portrayed science not as a linear accumulation of facts but as a dynamic process marked by paradigm shifts. This image closely relates with CT, which likewise requires questioning assumptions, evaluating anomalies, and revising beliefs in light of new evidence (Loving & Cobern, 2000; Tian et al., 2025). In the present study, Kuhn’s framework is adopted heuristically to foreground how anomalies destabilize prevailing standards of judgment and prompt qualitative reorganization of evaluative criteria, rather than to describe general cognitive development mechanisms. This emphasis aligns closely with our focus on questioning as a process that transforms multiple criteria under contradictory evidence. Scholars have noted that Kuhn’s ideas have been influential in science education, particularly in discussions of conceptual change and constructivist approaches (Matthews, 2024; Shan, 2020; W.-Z. Shi, 2015). Yet despite this influence, the explicit potential of Kuhn’s paradigm theory as a framework for cultivating students’ CT—especially within biology education—remains underexplored (Mengistie & Worku, 2020; Niaz, 2010). Connecting Kuhn’s account of scientific progress with students’ CT skills offers a useful theoretical direction for designing learning environments where learners engage in critique, reflection, and evidence-based revision. However, this integration has not been sufficiently developed in empirical research on classroom practice.

Against this background, the present study first develops a substantive theoretical framework for CT in biology education through qualitative analysis. Using students’ engagement with a “gene mutation” task—a context that involves conflicting evidence and conceptual uncertainty—the study identifies how learners confront anomalies, revise explanations, and negotiate conceptual change. Building on this empirically grounded framework, the research then draws an analogy to Kuhn’s cycle of normal science, anomaly, crisis, and paradigm shift. This comparison provides a methodological basis for incorporating history and philosophy of science (HPS) into practical pathways for fostering CT in biology classrooms (Cardinot et al., 2023; X. Shi, 2025). Accordingly, the study extends existing scholarship by examining a dimension that has not been fully explored: while Kuhn’s ideas have shaped research on conceptual change and constructivist pedagogy (Mengistie & Worku, 2020; Shan, 2020), their explicit application to CT development in subject-specific science education remains limited. By demonstrating how students’ CT processes can be understood through a Kuhnian lens, the research contributes both theoretically and practically to science education. It highlights the importance of embedding philosophical perspectives into biology teaching and offers a new approach to advancing students’ CT in meaningful and discipline-grounded ways.

## 2. Literature Review

### 2.1. Critical Thinking and Science Education

CT has long been recognized as a central competence for 21st-century learners, essential for problem solving, evaluating arguments, academic achievement, and personal growth (Altun & Yildirim, 2023). Its importance is widely accepted, yet its definition remains debated. Two broad perspectives can be distinguished. One view considers CT as a facet of intelligence, while another regards it as a set of teachable skills and dispositions (Willingham, 2008). Paul and Elder (2013) proposed a framework that integrates elements of thought, intellectual standards, and intellectual virtues to explain CT, emphasizing that high-quality thinking is guided by explicit standards and cultivated through intellectual virtues. However, some experts have challenged the view of CT as merely a collection of general intellectual skills, arguing that CT consists of specific forms of reasoning governed by rational standards and grounded in the evaluation of evidence (Ennis, 2015; Mulnix, 2012; Putra et al., 2023; Yan, 2021). Within this line of work, Ennis defined CT as “reasonable reflective thinking focused on deciding what to believe or do,” highlighting its general evaluative and decision-oriented function. In contrast, McPeck (1990) described CT as “the propensity and skill to engage in an activity with reflective skepticism,” emphasizing that CT is closely tied to the epistemic demands of particular domains and activities. These two definitions represent complementary perspectives: Ennis’s definition captures the broad, general purpose of CT, whereas McPeck’s conception underscores its context- and discipline-sensitive character.

The conceptualization of CT was further refined through a Delphi study conducted under the auspices of the American Philosophical Association (APA), led by Facione (1990). The Delphi definition was intended to provide a common reference point for research and assessment, in response to longstanding disagreements about the nature of CT. Drawing on rounds of expert consultation, the panel characterized CT as “purposeful, self-regulatory judgment” involving interpretation, analysis, evaluation, inference, and explanation. Importantly, the Delphi consensus further distinguished two interrelated dimensions of CT: cognitive skills and affective dispositions. CT skills include interpretation, analysis, evaluation, inference, explanation, and self-regulation, whereas CT dispositions refer to habitual tendencies such as truth-seeking, curiosity, open-mindedness, flexibility, honesty in confronting one’s own biases, and a willingness to reconsider (Facione, 1990; Hu & Bi, 2025). Together, these two dimensions suggest that CT involves not only what individuals are able to do cognitively, but also what they are habitually willing to do when confronted with problems or dilemmas. Nevertheless, while such consensus definitions provide a common ground, their generality raises questions about how CT should be taught and assessed across diverse subject domains.

One of the most enduring debates concerns whether CT should be regarded as a general transferable skill or a domain-specific competence (Abrami et al., 2015; Moeiniasl et al., 2022; Renaud & Murray, 2008). Generalist views assume that once learned, CT skills can be applied across contexts, while specifist views stress that CT depends on substantive knowledge and conventions within each discipline (Cáceres et al., 2020; Huang & Sang, 2023; McPeck, 1990; T. Moore, 2004; T. J. Moore, 2011). This debate has direct implications for pedagogy: if CT is context-sensitive, cultivating it requires attention to the epistemic structures, standards of evidence, and modes of reasoning particular to each subject. In other words, the lack of clarity between general and discipline-specific perspectives has complicated how educators design effective strategies to foster CT in classrooms.

To address these challenges, researchers have begun to propose structured frameworks that articulate CT in terms of distinct but interrelated components. For example, building on Facione’s (1990) Delphi report, subsequent studies have suggested that CT comprises at least three elements: skills (e.g., analysis, inference, evaluation), dispositions (e.g., curiosity, open-mindedness, willingness to reconsider), and background knowledge relevant to the subject domain (Davies, 2013; Hu & Bi, 2025). Such componential or multidimensional models have been validated in several contexts, including English as a Foreign Language and science education (Li & Liu, 2021; Tian et al., 2025). These efforts demonstrate the feasibility of operationalizing CT within specific educational domains.

However, despite these advances, biology education has received limited attention in this regard (McMurray et al., 1991; Mkimbili, 2024). Most existing models either address science education broadly or focus on other disciplines, leaving biology—a subject that inherently involves conflicting evidence, conceptual uncertainty, and socioscientific issues—less explored (Bailin, 2002; García-Carmona, 2025; Hu & Bi, 2025). Recent attempts to construct science-specific CT frameworks through Delphi consensus and assessment tools highlight both the feasibility and necessity of such endeavors (Tian et al., 2025), yet few studies have addressed how such frameworks might be developed for biology classrooms in particular. This study therefore seeks to contribute to this underexplored area by constructing a theoretical framework of CT grounded in students’ engagement with biological concepts, thereby offering insights into how CT can be cultivated within domain-specific contexts.

### 2.2. Kuhn’s Structure of Scientific Revolutions and Critical Thinking

Thomas Kuhn’s The Structure of Scientific Revolutions offered a nonlinear description of scientific development, emphasizing that progress does not occur through the steady accumulation of facts but through paradigm shifts (Kuhn, 1962; Wray, 2011). Specifically, when persistent anomalies undermine the stability of normal science, a paradigm shift reorganizes theory and practice. In this cycle—normal science, anomaly, crisis, and revolution—scientific knowledge is understood as provisional, and the adoption of a new paradigm leads to a reorganization of both theory and practice (Matthews, 2024; Siegel, 2004). Previous studies have shown that Kuhn’s vocabulary and ideas have been widely applied in science education discourse, particularly around conceptual change, the nature of science (NOS), and constructivist perspectives (Niaz, 2010; W.-Z. Shi, 2015).

The educational significance of Kuhn’s framework has been widely discussed. Matthews (2004) argued that presenting science as a process of shifting paradigms helps students see science as developing rather than static, and later reflections emphasized that Kuhn’s work continues to shape how the NOS can be introduced in curricula (Matthews, 2024). Work in curriculum and teacher education has highlighted the importance of exposing the presuppositions and controversies often hidden in textbook accounts of “finished” science; such discussions recommend using historical cases to show how anomalies and competing explanations were addressed in practice (Niaz, 2010). Kuhn’s concepts of presuppositions and paradigm commitments can also deepen teachers’ awareness of how scientific concepts are situated historically and philosophically (Arabatzis & Kindi, 2008; Schindler, 2013). Studies have further shown that teaching strategies inspired by Kuhn’s insights—such as engaging learners with historical scientific controversies—can foster deeper reflection on NOS (Mengistie & Worku, 2020; Oh, 2017). This is particularly relevant for cultivating CT, as evaluating evidence, questioning assumptions, and revising explanations mirror the processes that occur during scientific crises (Matthews, 2004; Siegel, 2004). Overall, these discussions show that Kuhn’s philosophy encourages educators to emphasize the tentativeness of knowledge, the role of anomalies, and the historical context of science.

The educational applications of Kuhn’s framework connect closely with CT. Just as scientists must confront anomalies that challenge accepted theories, students must also engage with contradictory evidence that unsettles prior conceptions, question assumptions, and revise explanations when needed. The movement from stable understanding to cognitive conflict and then to restructured concepts parallels the trajectory from normal science, to crisis, and then to a new paradigm. This analogy can be used to design classroom tasks that encourage the evaluation of competing claims and reflection on standards of argumentation (Mengistie & Worku, 2020; Siegel, 2004). By engaging with scenarios of scientific crisis—such as the replacement of Newtonian mechanics by relativity or debates about genetics and inheritance—students can experience how doubt and conflict stimulate reasoning and reflection, which are central processes of CT (Matthews, 2024). At the same time, recent HPS scholarship has urged educators to approach Kuhn with care—clarifying what his model implies and does not imply (e.g., avoiding overgeneralized relativism) and updating classroom narratives in light of later debates on paradigms, incommensurability, and exemplars (Matthews, 2024; Shan, 2020).

Despite its wide influence, the systematic application of Kuhn’s paradigm theory to subject-specific CT instruction—especially in biology—remains underexplored (Liu et al., 2025a). While Kuhn has often been cited to justify attention to NOS and conceptual change, few studies have traced specific teaching strategies from Kuhn’s paradigm theory into classroom practice or assessed their impact on students’ CT in particular domains (Mengistie & Worku, 2020). The present study analogizes students’ CT processes in core biology tasks through the theoretical lens of Kuhn’s paradigm shifts, analyzing how learners identify anomalies, manage conceptual tensions, and reconstruct explanations based on evidence. In doing so, it links HPS-based design principles with observable CT practices in biology (Cardinot et al., 2023; Liu et al., 2025a), thereby addressing a gap in the literature and offering a framework for integrating philosophical perspectives into disciplinary CT instruction.

## 3. Materials and Methods

### 3.1. Research Design

This study adopted a qualitative analytic design informed by the coding procedures articulated by Corbin and Strauss (2015). The primary objective of the present study was to develop an explanatory, discipline-specific theoretical model of CT in senior secondary biology through selective coding and theory integration. Rather than generating additional descriptive categories, the study focused on identifying a core category and explicating relationships among analytic categories to explain how students engage in questioning, reasoning, and evaluative-criteria transformation when confronted with contradictory evidence.

The analytic design builds on a multi-phase research program. In an earlier phase of the project, pen-and-paper responses and classroom dialogue transcripts were collected and analyzed to generate an initial codebook, major categories, and memos. These memos documented coding decisions and category development and served as analytic records for subsequent theory integration. The first-cycle analyses based on this corpus have been reported elsewhere (Liu et al., 2025b). These previously coded materials provide an analytic foundation for the present study.

In the present study, a new dataset of cross-regional pen-and-paper responses was collected specifically to support selective coding and theoretical model construction. These responses were randomly assigned, using a reproducible procedure, to four analytic batches. Batches A–C were introduced sequentially during selective coding to support constant comparison and iterative refinement of category properties and relationships. Batch D was withheld until the codebook was stabilized and was then analyzed solely for external validation.

**Figure 1:**
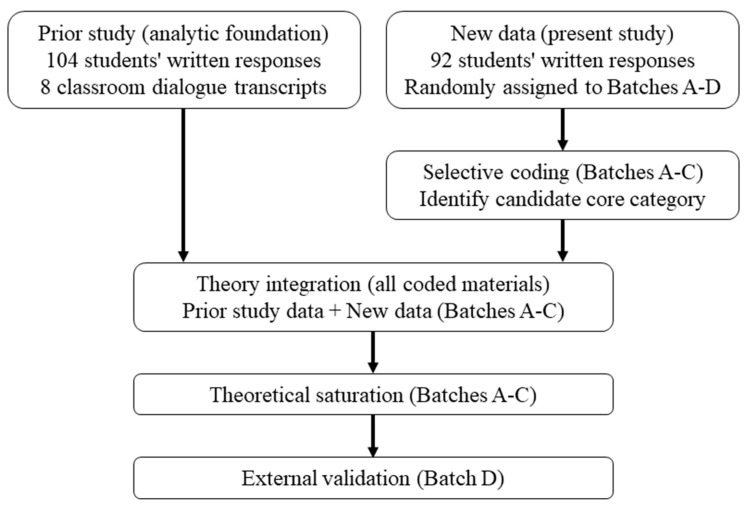
The study process.

During theory integration, all available coded materials were consulted (Figure 1). The earlier corpus was not subjected to a new round of open or axial coding; rather, it was used to examine the stability, scope, and coherence of category relationships identified through selective coding of the new dataset.

Throughout the analytic process, constant comparison and memo writing guided category refinement and core-category identification. Earlier memos were retained to document the evolution of analytic decisions, while new memos recorded how additional cases confirmed or challenged emerging relationships. Theoretical saturation was assessed by monitoring whether successive cases added new properties, dimensions, or relationships. All analytic decisions were documented through versioned codebooks and dated memos to maintain an audit trail. The study received institutional ethics approval, and informed consent was obtained from all participants.

### 3.2. Participants and Context

In this study, convenience sampling was employed to recruit 92 11th–grade students (ages 15–17; 48 male, 44 female) from a comprehensive high school in Northern China. All participants were enrolled in biology courses aligned with the national curriculum standards. Participation was voluntary, with consent obtained in accordance with institutional ethical guidelines, and all responses were anonymized prior to analysis.

In an earlier phase of the same research project, 104 students completed pen-and-paper responses, and eight students participated in classroom dialogue activities.

### 3.3. Instrument

**Figure 2:**
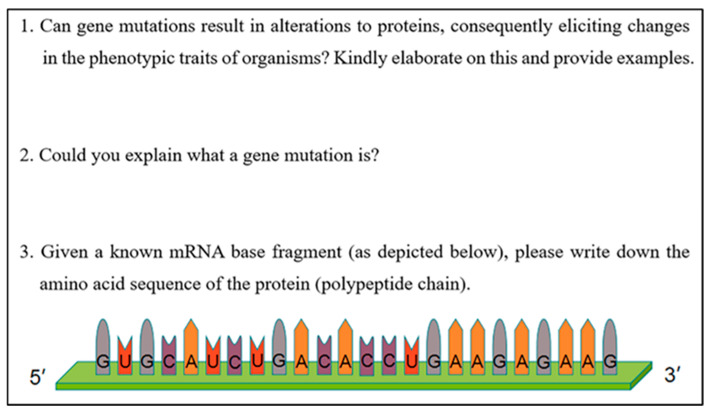
The pen-and-paper test tool (reproduced from Liu et al. (2025b)).

An additional dataset was collected using the same pen-and-paper instrument as in the prior study. The instrument comprised three open-ended prompts addressing gene mutation, protein change, and phenotypic traits (Figure 2) and was designed to elicit students’ thinking processes as they constructed written explanations. Although the prompts did not embed evidential materials directly, the first prompt explicitly required students to consider both supportive and conflicting evidence, thereby encouraging evidential reasoning.

The first question asked, “Do genetic mutations lead to changes in organism traits?” Possible responses included “Yes,” “No,” “It depends; genetic code degeneracy will not alter the trait, but other cases will,” and “I don’t know.” The task was structured to differentiate between responses that assume a deterministic causal chain linking mutation, protein change, and phenotype change and responses that recognize cases of genetic code degeneracy, in which base substitutions do not lead to amino-acid changes and therefore do not result in phenotypic change.

Examples of mutations that change amino-acid sequences and phenotypes are more common in textbooks and classroom instruction than cases involving genetic code degeneracy. Within this instructional context, genetic code degeneracy functions as a contradictory case that makes it possible to observe how students respond to anomalous evidence and whether they shift from confirmation toward questioning and potential falsification. Accordingly, genetic code degeneracy serves as the central anomalous case in the design of the instrument.

### 3.4. Data Collection

The empirical corpus comprised two sets of qualitative materials, including data collected in a prior study and data newly collected in the present study. Materials from the prior study consisted of 312 pen-and-paper responses produced by 104 students using a pen-and-paper test instrument and eight classroom dialogue transcripts generated from students who participated in a falsification-heuristic activity (Liu et al., 2025b).

In the newly present study, the same pen-and-paper instrument was administered to 92 participants, yielding 276 written responses (three per student). All responses were collected during regular biology class sessions and anonymized prior to analysis. Accordingly, the corpus consisted of written responses from 196 students (104 from the prior study and 92 newly collected), together with classroom dialogue data from eight students.

### 3.5. Data Analysis

Data analysis integrated previously collected and newly gathered materials within a shared coding framework. The previously collected corpus provided a validated codebook and category system derived from open and axial coding, whereas the newly collected data primarily supported selective coding. Both datasets were subsequently consulted during theory integration.

Inter-coder agreement had been established in the earlier phase of the project during first-cycle coding, using the same coding team and procedures reported in Liu et al. (2025b). Therefore, inter-coder reliability was not recalculated for the present selective-coding analysis. The coding team consisted of a secondary-school biology teacher with over ten years of teaching experience who is also a doctoral student, and another doctoral student in science education. Both coders have biology-related academic backgrounds, are familiar with gene mutation content, and have prior experience with qualitative coding in science education research.

#### 3.5.1. Selective Coding and Core Category

Using Batches A–C of the newly collected written responses, selective coding proceeded from validated analytic categories (e.g., reasoned denial/affirmation, result-focused judgment, causal/abductive/deductive/inductive reasoning, and criteria in use), which were documented in earlier memos. These memos specified how students’ judgments (“not necessarily,” “will,” “will not”) were coupled with supporting reasons, often expressed through conditional structures (“if… because… therefore…”) or through the organization of knowledge units and logical partitions. During selective coding, these memos functioned as decision rules for category consolidation and boundary setting.

Written responses were segmented into meaning units representing judgments, reasons, or explanatory moves, and these units were coded using the validated category system. During this process, newly collected data were constantly compared with the existing codebook to examine whether previously identified categories remained applicable and sufficient.

Guided by a memo, questioning and transforming multiple criteria was treated as the candidate core process because it linked students’ handling of supportive versus conflicting evidence with shifts in the criteria applied (from authority-based to internalized, reconstructed criteria) (see Appendix B). Selective coding examined whether this process saturated relationships with: (a) forms of reasoning, (b) analytic skills, and (c) the stability or fragility of judgment criteria. Core-category adjudication followed three criteria: centrality, explanatory power across tasks, and growth potential.

#### 3.5.2. Theory Integration

After a preliminary core category and major category relationships were identified through selective coding, theory integration was conducted. At this stage, all available coded materials—including the previously collected 104 written responses, the eight classroom dialogues, and the newly collected written responses from Batches A–C—were consulted to examine the coherence, scope, and stability of the emerging explanatory model. The previously collected corpus was not subjected to a new round of open or axial coding; instead, it was used to check whether the category relationships identified through selective coding could also account for patterns observed in earlier data.

Classroom dialogues were particularly useful for identifying moments of questioning and responses to conflicting evidence, because such processes unfold temporally and are not always explicit in written responses. Written responses can also contain indications of questioning and attention to conflicting evidence, but they primarily supported identification of judgment patterns, reasoning forms, and criteria in use.

#### 3.5.3. Theoretical Sampling & Rolling Comparison

During selective coding, Batches A–C of the newly collected written responses were sequentially processed in an analytic loop to support theoretical sampling and rolling constant comparison. As additional cases were analyzed, data were constantly compared with the existing codebook and existing category relationships to examine their applicability, sufficiency, and stability. This process allowed the study to test whether previously identified categories remained adequate, whether the candidate core category continued to account for new cases, and whether relationships among categories required refinement.

The criteria to proceed to the next analytic pass were: (i) no new properties emerging in two consecutive passes; (ii) all relationship hypotheses from the memo tested at least once.

#### 3.5.4. Theoretical Saturation

Theoretical saturation was defined as the point at which (a) no new properties or dimensions were added to selective-coding categories across a full analytic pass, and (b) proposed relationships among questioning, criteria, reasoning form, and analytic skills could be explained entirely by existing codes and memos. Saturation judgments were based on continued analysis of Batches A–C. Analytic decisions related to saturation were recorded in a running log (see Appendix C).

#### 3.5.5. External Validation

After categories and linkages were stabilized, Batch D, consisting of the newly collected written responses, was exclusively analyzed for external validation (see Appendix D). The purpose of this step was to examine whether the established categories and relationships could account for cases not previously used in selective coding. We sought both confirming evidence and disconfirming evidence. Where Batch-D instances diverged, we documented the variance as limits to transferability rather than modifying categories; only if multiple, independent disconfirmations targeted the same linkage would we reopen the codebook and flag any revision in the audit table. In the present study, no instances in Batch D triggered reopening of the codebook or modification of existing categories. Table 1 presents the finalized codebook.

## 4. Results

### 4.1. The Theoretical Model of Critical Thinking

Analysis of students’ written responses and classroom dialogues focused on how learners handled supportive and conflicting evidence in a gene-mutation context and how their judgments, reasoning, and evaluative criteria changed across these situations. During selective coding, cases from Batches A–C were examined through constant comparison, and memos were used to explore which processes appeared central for organizing relationships among categories.

Across cases, moments of questioning consistently co-occurred with shifts in the criteria students used to evaluate evidence. When students encountered results that contradicted their initial expectations, some simply appended conditions or ignored the anomaly, whereas others began to question their original assumptions and reconsider what counted as acceptable evidence or explanation. Alternative candidate processes (e.g., explanation, comparison, or hypothesis generation) were observed, but these appeared to depend on whether questioning was triggered. On this basis, questioning and transforming multiple criteria was identified as the core category because it accounted for how different forms of reasoning, analytic actions, and judgment patterns became coordinated when students attempted to restore coherence in the face of contradiction.

**Figure 3:**
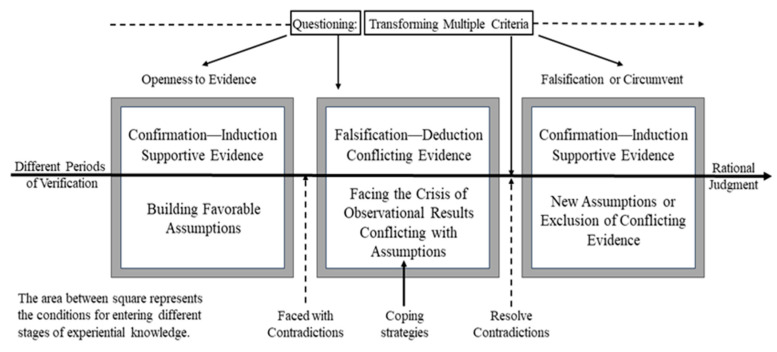
The theoretical model of critical thinking in biology.

Four major categories were found to relate systematically to this core category: evolving evaluative criteria, various types of reasoning, analysis without judgment, and the application of empirical knowledge to different criteria. Together, these categories describe a dynamic process in which students move between confirmation and falsification, adjust the standards by which they judge evidence, and reconstruct hypotheses accordingly. Figure 3 presents the theoretical model derived from this integration.

At an early stage of problem solving, students’ judgments are typically guided by evaluative criteria shaped by prior instruction and everyday experience. These criteria may support relatively stable expectations (e.g., “mutation changes protein, therefore changes phenotype”) and lead students to seek supportive evidence. When contradictory evidence appears, however, these criteria are challenged. Some students respond by questioning their initial assumptions and reconsidering what counts as valid evidence or explanation, whereas others retain their original criteria and attempt to preserve their conclusions. Three recurrent judgment patterns were identified in students’ written responses: “Yes,” “No,” and “It depends; genetic code degeneracy will not alter the trait, but other cases will.” The first pattern relied on a deterministic causal chain and ignored genetic codon degeneracy; the second rejected phenotypic change but did not attend to evidence that many mutations alter amino acid sequences and phenotypes; the third integrated both lines of evidence. Constant comparison showed that the core process emerged only when students questioned the tension between these competing pieces of evidence and reconstructed evaluative criteria. Accordingly, questioning and transforming multiple criteria was identified as the core category.

The model therefore conceptualizes CT in this context as a cyclical process in which students move between supportive and conflicting evidence, engage in questioning, and transform multiple evaluative criteria. Through this process, hypotheses are not simply confirmed or rejected but are reconstructed on the basis of revised standards of judgment.

#### 4.1.1. Evolving Evaluative Criteria

Evolving evaluative criteria refers to changes in the standards that students use to judge evidence, explanations, and hypotheses when they encounter problem situations involving supportive or conflicting evidence. Rather than relying on a single fixed criterion, students shift, extend, or reconstruct their criteria in order to make sense of unexpected results and to decide whether a hypothesis should be maintained, modified, or rejected.

This category is closely connected to the core process of questioning and transforming multiple criteria. When questioning is triggered by contradictory evidence, students begin to reconsider what counts as acceptable evidence and what constitutes a satisfactory explanation. Through questioning, previously stable criteria may become unstable and are replaced or supplemented by new, more conditional criteria. In contrast, when questioning does not occur, students tend to retain their original criteria and attempt to preserve their initial judgments.

**Figure 4:**
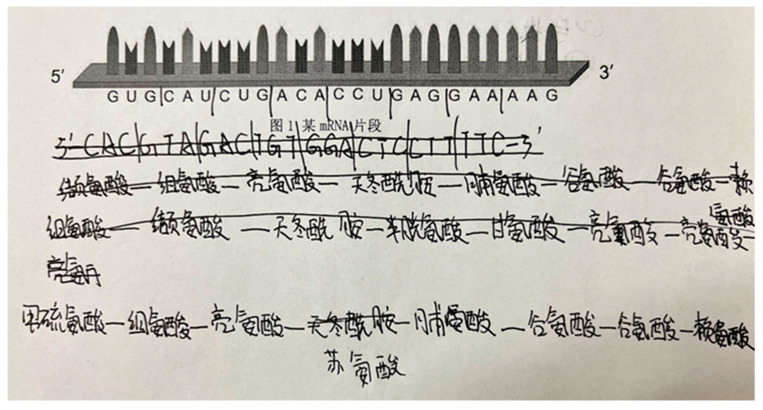
Evolving Evaluative Criteria in Case 47 (Batch A), Question 3. Note. The amino acid names in the figure are presented in Chinese to preserve the authenticity of students’ original written responses. Their corresponding English names and three-letter abbreviations are provided as follows: 缬氨酸 (Valine, Val), 组氨酸 (Histidine, His), 亮氨酸 (Leucine, Leu), 天冬酰胺 (Asparagine, Asn), 脯氨酸 (Proline, Pro), 谷氨酸 (Glutamic acid, Glu), 赖氨酸 (Lysine, Lys), 半胱氨酸 (Cysteine, Cys), 甘氨酸 (Glycine, Gly), 甲硫氨酸 (Methionine, Met), 苏氨酸 (Threonine, Thr), 图1 某mRNA片段 (Figure 1. A fragment of the mRNA).

For example, in Figure 4 (Case 47, Batch A), the participant initially treated the provided mRNA fragment as if it were a DNA sequence and first performed transcription before translation. This response indicates an unstable evaluative criterion regarding sequence identification, as the student failed to distinguish between mRNA and DNA. Nevertheless, the student translated the resulting codons (e.g., CAC…TTC) by applying the principle of complementary base pairing and the genetic code, suggesting that their procedural biological knowledge was stable and that their reasoning followed a rule-based, deductive pattern.

Subsequently, the student crossed out the transcribed and translated amino acid sequence and initiated a new translation process directly from the given mRNA fragment, ultimately generating a correct amino acid sequence beginning with methionine and ending with lysine. This revision reflects questioning of the initial procedure, which in turn triggered a transformation of the evaluative criterion—from “any nucleic-acid sequence requires transcription before translation” to “mRNA can be directly translated.” After this shift, the student’s reasoning and judgment aligned with biologically appropriate standards.

This case illustrates that evaluative criteria are not static but are progressively reshaped as students engage with conflicting evidence. Within the model, such evolution of criteria provides the basis for deeper questioning and supports movement between confirmation and falsification, thereby enabling the reconstruction of hypotheses.

#### 4.1.2. Various Types of Reasoning

Various types of reasoning refer to the inferential patterns students employ when constructing, evaluating, and revising hypotheses in evidence-based problem situations. Within the model, reasoning functions as the cognitive means through which students connect evidence, prior knowledge, and emerging evaluative criteria, thereby supporting judgment and hypothesis construction. In these moments, students use different reasoning patterns to coordinate evidence with the evaluative criteria they currently hold, and, in some cases, to reconsider those criteria.

When students marshal multiple pieces of evidence or appeal to biological principles to support a claim, they draw on different types of reasoning to coordinate evidence with the evaluative criteria they currently hold. Patterns in the data suggest that the choice of reasoning is closely related to how confident students are in their judgments and how stable their evaluative criteria are. When students experience uncertainty or partial doubt about their own conclusions, they tend to rely on inductive, abductive, or causal reasoning to reconcile available evidence with provisional criteria. In contrast, when students invoke general biological principles to justify a claim, they more often employ deductive reasoning, indicating the presence of relatively stable and internalized evaluative criteria.

*Not necessarily. (1) Sickle-cell anemia is caused by gene mutation, which leads to amino acid substitution in the peptide chain forming hemoglobin, and thus results in changes in organism traits (Abductive reasoning).*

*(2) Because of genetic codon degeneracy, one amino acid may correspond to several codons. When one or several of them are changed, the amino acid may not change (Deductive reasoning) (Case 33, Batch C).*

*It may cause change. If the mutation is located in an intron, it will not cause protein change; but if it is located in an exon, it will cause protein change (Causal reasoning) (Case 60, Batch B).*

*It may or may not. If a change or substitution occurs in a non-coding region and the expressed amino acid does not change, then no trait change occurs, such as GAG changing to GAA, which both encode glutamic acid; if bases are inserted, deleted, or substituted in a coding region and the amino acid changes, then it causes change, such as sickle-cell anemia (Inductive reasoning) (Case 21, Batch A).*

For example, in Case 21 (Batch A), the participant answered that gene mutation may or may not lead to phenotypic change, explicitly presenting two possible outcomes. This response suggests uncertainty about a single definitive conclusion. To support this position, the student used multiple pieces of evidence, referring to both coding and non-coding regions as well as synonymous and nonsynonymous substitutions. This pattern reflects inductive reasoning, in which several specific situations are aggregated to justify a conditional generalization.

Notably, however, the student did not explicitly invoke the principle of codon degeneracy to explain why some substitutions do not change amino acids. Instead, the participant relied on assembling multiple examples to compensate for this gap. This suggests a degree of tentative confidence in the judgment: the student appears aware that exceptions exist but lacks a stable conceptual criterion to explain them. In this sense, the inductive accumulation of evidence functions as a way to manage uncertainty and to fill perceived explanatory gaps.

By contrast, the participant who answered “not necessarily” in Case 33 (Batch C) directly cited genetic codon degeneracy, stating that one amino acid may correspond to several codons and that changing one or several codons does not necessarily change the amino acid. This represents deductive reasoning, as a general biological principle is applied to evaluate a specific claim. Although this participant did not overtly revise their evaluative criteria during the response, the use of this principle indicates a relatively stable and biologically appropriate standard for judging the relationship between mutation and phenotype.

These cases illustrate that different reasoning patterns reflect different degrees of stability in evaluative criteria. Inductive reasoning may signal tentative understanding supported by multiple examples, whereas deductive reasoning grounded in disciplinary principles reflects more consolidated criteria for evaluating contradictory evidence.

#### 4.1.3. Analysis Without Judgment

Students often engage in analysis before making any explicit judgment. They attend to features of the data, organize information, and understand and compare relationships among different features and pieces of information, even when they have not yet evaluated correctness or plausibility. Such analysis supports the organization of evidence and creates conditions for the later application, questioning, or revision of evaluative criteria.

Across cases, analysis is reflected in students’ identification of information units, classification of elements, segmentation of sequences, and comparison between symbolic representations and known conventions or standards. When students encounter discrepancies, these analytical operations help localize where a potential problem exists. However, analysis alone does not guarantee that students will question their existing criteria. Only when comparison highlights a mismatch between the standards students hold and the situation they encounter does questioning emerge, which may then lead to judgment or transformation of evaluative criteria.

**Figure 5:**
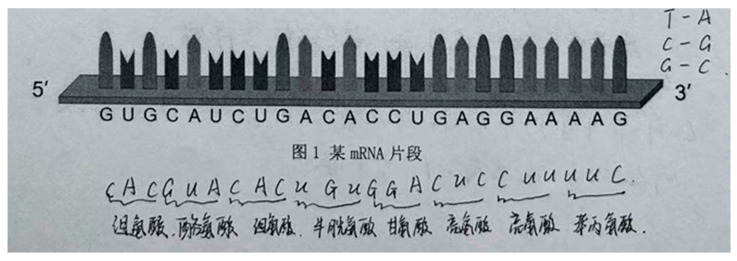
Analysis Without Judgment in Case 34 (Batch A), Question 3. Note. The amino acid names corresponding English names and three-letter abbreviations are provided as follows: 组氨酸 (Histidine, His), 酪氨酸 (Tyrosine, Tyr), 半胱氨酸 (Cysteine, Cys), 甘氨酸 (Glycine, Gly), 亮氨酸 (Leucine, Leu), 苯丙氨酸 (Phenylalanine, Phe).

For example, in Figure 5 (Case 34, Batch A), the participant initially treated the provided mRNA fragment as if it were a DNA sequence and performed transcription, generating a sequence such as CACGUA…AAG. This action indicates that the student applied an existing evaluative criterion for sequence processing without recognizing that the initial identification of the sequence type was incorrect. At this stage, no questioning or decision-making occurred.

At the same time, the participant marked every three bases as a unit, reflecting the classification of bases into codons. This segmentation shows that the student organized the sequence according to a familiar structural rule, even though the criterion guiding the procedure remained inappropriate.

A contrast can be seen in Figure 4, where a participant crossed out an initially translated amino acid sequence and initiated a new translation process. This action suggests that comparison between different amino acid translates made the participant aware that the mRNA sequence should be directly matched to the codon table. Here, comparison created a condition in which questioning became possible and a new judgment was triggered.

Therefore, analysis such as identification, classification, and comparison do not themselves constitute questioning or decision-making, but they provide the structural basis upon which discrepancies can be noticed. Whether analysis leads to questioning depends on whether students interpret the detected mismatch as meaningful. In this way, analysis supports but does not determine the transformation of evaluative criteria.

#### 4.1.4. Application of Empirical Knowledge to Different Criteria

Students’ empirical knowledge, derived from prior instruction and accumulated learning experiences, plays a central role in shaping how evaluative criteria are constructed, maintained, and transformed during evidence-based problem solving. Rather than functioning as isolated factual recall, empirical knowledge is mobilized as a resource for interpreting evidence, selecting reasoning strategies, and determining which standards are appropriate for judging hypotheses in a given situation.

Empirical knowledge becomes consequential when students encounter discrepancies between expected and observed outcomes. At this point, questioning may be triggered, prompting students to reconsider how their existing knowledge should be applied and whether alternative standards are needed. In other words, empirical knowledge does not directly produce questioning; instead, questioning reorganizes how empirical knowledge is used, allowing certain pieces of knowledge to be reinterpreted as new evaluative criteria.

This pattern is clearly visible in Figure 4 (Case 47, Batch A), which has previously been analyzed in relation to evolving evaluative criteria and analysis without judgment. The participant initially applied the familiar textbooks-based procedure of transcription followed by translation, indicating reliance on a routine piece of empirical knowledge about gene expression. However, after recognizing a mismatch between the translated result and expectations, the student abandoned the transcription step and directly translated the given mRNA fragment, drawing on a different piece of empirical knowledge—namely, that mRNA sequences correspond directly to codons in the genetic code. In this case, empirical knowledge was not replaced but reorganized: questioning enabled the student to privilege a more context-appropriate standard for applying biological knowledge.

A similar pattern appears in Case 21 (Batch A), where the student stated that gene mutation “may or may not” lead to phenotypic change and supported this position by referring to multiple situations, including coding versus non-coding regions and synonymous substitutions. The student drew on several fragments of empirical knowledge but did not explicitly invoke codon degeneracy as an organizing principle. Instead, empirical knowledge was applied in an example-based manner, allowing the student to manage uncertainty without fully transforming evaluative criteria.

By contrast, in Case 33 (Batch C), the participant directly invoked genetic codon degeneracy to explain why some base substitutions do not change amino acids. This response reflects a more consolidated application of empirical knowledge, where a general biological principle is used as a stable standard for evaluating the mutation–phenotype relationship. Although no overt questioning is observed, the knowledge is flexibly applied to accommodate potentially contradictory evidence, indicating that empirical knowledge can also sustain stable evaluative criteria when it is well integrated.

Across these cases, empirical knowledge both constitutes part of students’ evaluative criteria and provides the substantive content of their reasoning, whereas questioning that transforms multiple criteria determines how empirical knowledge is selected, reorganized, and brought into relation with evidence in support of judgment.

### 4.2. Theoretical Convergences: Kuhn’s Paradigm Shifts and the Process of Critical Thinking

This section interprets the model of CT through the lens of Kuhn’s philosophy of science, drawing on both written responses and classroom dialogues to illuminate how students engage in questioning, reasoning, and evaluative-criterion use over time. By aligning students’ reasoning with the cycle of normal science, anomaly, crisis, and scientific revolution, the analysis highlights structural parallels between the development of scientific knowledge and the cognitive shifts that occur when learners confront contradictory evidence. It must be emphasized, however, that the use of Kuhn’s theory here is heuristic and limited: Kuhn’s account of scientific revolutions provides an interpretive analogy rather than a direct mapping of classroom cognition (Matthews, 2004; Shan, 2020). Such a dialogue underscores how anomalies function as catalysts for both scientific revolutions and CT, offering a deeper theoretical understanding of the dynamic processes that drive cognitive growth in educational contexts.

#### 4.2.1. The Cycle of “Normal Science” and the Confirmation Phase in Critical Thinking

Kuhn’s notion of normal science—where scientists operate within a dominant paradigm by refining theories through puzzle-solving—bears a strong structural resemblance to the confirmation phase of CT. In both contexts, reasoning is guided by established criteria: scientists follow paradigm-based exemplars, while students rely on prior knowledge to validate hypotheses through inductive reasoning.

**Figure 6:**
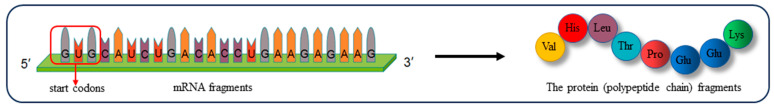
The student’s identification and reasoning process (Case 84, Batch C).

This parallel was clearly reflected in students’ written responses. When participants translated an mRNA sequence into a polypeptide using the codon table, many successfully confirmed their original hypotheses by applying stable evaluative criteria. For example, one student (Case 84, Batch C, Question 3) correctly identified start codons and produced a complete amino acid chain, demonstrating that prior conceptual frameworks could reliably guide problem-solving (Figure 6). This reasoning exemplifies Kuhn’s description of normal science, where anomalies are initially excluded and consistency within the paradigm is prioritized.

Thus, both in Kuhn’s science and in students’ CT, the early stage of inquiry emphasizes stability and coherence, aiming to extend the explanatory scope of existing frameworks without questioning their foundations.

#### 4.2.2. Anomaly as Cognitive Disruptor: Unpacking the “Crisis” Analogy in Critical Thinking

In Kuhn’s analysis, anomalies disrupt normal science when persistent contradictions can no longer be dismissed as exceptions, leading to a crisis. The same logic applies to CT: when contradictory evidence confronts established evaluative criteria, students experience cognitive dissonance that forces a reevaluation of criteria.

A striking case arose in classroom dialogues task. A participant initially assumed that a base substitution would necessarily alter the amino acid sequence. Yet, upon translation, the amino acid chain remained unchanged. The student then explained “Because A was replaced by U, but both codons encode glutamic acid. This is due to the degeneracy of the genetic code.” This moment of recognition mirrors Kuhn’s anomaly as the empirical result could not be integrated into the initial hypothesis, compelling the learner to confront the limits of prior criteria. For instance,

Teacher: “Which type of gene mutation corresponds to the second mRNA sequence?”

Student: (Comparing with the normal sequence) “Substitution.”

Teacher: “Does this gene mutation result in a change in the amino acid sequence?”

Student: “No.”

Teacher: “Can you explain why this is the case?”

Student: “Because the A base is replaced by a U base, but both codons correspond to the same amino acid, glutamic acid. Since genetic codons exhibit degeneracy, the amino acid sequence remains unchanged”.

(participant #04)

Such crises loosen the grip of routine reasoning, much as Kuhn argued that scientific crises relax the rules of normal practice and open the way for revolutionary change. In CT, too, the confrontation with anomalies shifts students from merely affirming supportive evidence to actively questioning conflicting data. This transition illustrates that cognitive growth, like scientific progress, emerges not from smooth accumulation but from disruptive encounters that redefine criteria of evaluation.

#### 4.2.3. Incommensurability and the Reconstruction of Evaluative Frameworks

Kuhn’s concept of incommensurability—the inability to fully translate concepts across paradigms—offers a powerful analogy for how students reconstruct evaluative frameworks when faced with contradictory evidence. Just as the transition from Newtonian mechanics to relativity redefined fundamental categories, learners in our study had to reorganize their criteria of judgment when anomalies challenged their initial assumptions.

A classroom episode illustrates this rupture. One student first identified a mutation as a deletion. However, after translating the mRNA and comparing it with the expected amino acid sequence, the student corrected themselves: “It is not a deletion; it’s a substitution.” This shift reflected more than error correction; it demonstrated a reorganization of evaluative criteria. The learner abandoned the original standard of “missing bases must equal deletion” and adopted a new standard based on direct comparison of translated products. For instance,

Teacher: “Which type of gene mutation does this transcribed fragment represent?”

Student: “Deletion.”

Teacher: “What consequence does the deletion produce?”

Student: “No termination.”

Teacher: “Compared with the normal amino acid sequence, is there any change?”

Student: “No—(revises) it’s a substitution.”.

(participant #01)

Such moments exemplify Kuhn’s notion of a “Gestalt switch.” Old frameworks cannot simply be patched; they must be restructured. In CT, evaluative criteria—whether inductive or deductive, causal or probabilistic—are not additively expanded but qualitatively transformed. This process aligns with Kuhn’s view that scientific revolutions require abandoning entrenched taxonomies to adopt new categorical schemes. Here again, the analogy is heuristic: students’ cognitive reorganizations resemble, but are not identical to, scientific paradigm shifts (Matthews, 2004, 2024).

#### 4.2.4. Paradigm Shifts and the Emergence of New Hypotheses

Kuhn argued that paradigm shifts reorganize the scientific landscape, retaining some explanatory power of the old while integrating anomalies into a new framework. The same pattern was observed in students’ reasoning: after grappling with contradictory evidence, they did not merely adjust prior hypotheses but reconstructed them to incorporate previously conflicting results.

In one case, after repeated experiments with different mutation types, a participant concluded: “Not every mutation changes the phenotype. Some substitutions leave the amino acid sequence unchanged because of genetic codon degeneracy.” Here, the student generated a new hypothesis that was both broader and more nuanced than the original assumption. This outcome illustrates how anomalies catalyze the emergence of integrative criteria, mirroring Kuhn’s description of revolutions as non-cumulative leaps. For instance,

Facilitator: Can gene mutations cause changes in an organism’s traits?

Student: Yes.

……

Facilitator: Now that all mRNA fragments from gene mutations have been examined, can you summarize your conclusions?

Student: The insertion, deletions, and substitutions in base fragments may influence the expression of traits.

Facilitator: It seems you’ve divided this into different scenarios. How do you view these cases?

Student: The base sequence was substituted to change the codon. However, different codons may express the same amino acid, which does not affect gene expression. However, deletions and insertions in the base sequence change amino acids and affect gene expression.

The integrative nature of these new hypotheses resonates with the core category we identified—questioning and transforming multiple criteria. Students shifted from inductive confirmation, through deductive falsification, and back to revised induction, reorganizing evaluative criteria at each stage. Their reasoning thus paralleled Kuhn’s cycle of normal science → anomaly → crisis → new paradigm.

In both science and CT, progress lies not in linear accumulation but in periodic reconfiguration. The selective retention of valid prior knowledge—such as the recognition that some mutations do change phenotypes—ensures continuity, while the adoption of new evaluative frameworks enables growth.

Taken together, these convergences suggest that Kuhn’s philosophy offers an illuminating yet limited metaphor for interpreting students’ CT. The analogy highlights that anomalies can drive learners to abandon inadequate evaluative criteria and reconstruct new ones, much as anomalies propel scientific revolutions. Importantly, Kuhn’s framework is used here to emphasize qualitative restructuring of evaluative standards, rather than merely the assimilation of additional information. For science educators, this underscores the pedagogical value of deliberately introducing contradictory evidence to trigger cognitive conflict and foster deeper questioning. As Matthews (2024) and Niaz (2010) note, such instructional use of anomalies can strengthen students’ understanding of NOS and enhance their capacity for critical evaluation.

## 5. Discussion

Building on case-based analyses of students’ written responses and classroom dialogues, this study articulates a discipline-specific explanatory model of students’ CT in senior secondary biology, centered on the core process of questioning as the driver of multiple criteria transformation under contradictory evidence. Rather than offering another broad definition or solely an assessment framework, the contribution here is a process explanation of how learners shuttle between confirmation and falsification, reopen or revise evaluative criteria, and recompose hypotheses in context. By specifying how criteria, reasoning forms, and analysis moves interact in a concrete biological task space, the model extends current CT study—which often concentrates on consensus definitions, generic skills/dispositions, or cross-disciplinary pedagogy. This addresses persistent calls to clarify what CT looks like within a discipline and under what conditions it develops (Hu & Bi, 2025; Li & Liu, 2021; Tian et al., 2025).

A long-standing debate pits generalist conceptions of CT against discipline-specific accounts. While general frameworks (e.g., Delphi/Facione) have been valuable for establishing shared terminology, they risk under-specifying the role of domain knowledge and evaluative criteria in actual reasoning episodes. Our findings extend specifist arguments by showing that the stability or fragility of criteria in use (e.g., correct rules for DNA/RNA bases) is pivotal for whether learners can transition from confirmation to falsification and back again—an aspect often implicit in generic models. This finding aligns with Paulsen and Kolstø (2022), who argue that students frequently base their reasoning on assumptions derived from initial hypotheses and often rely on assumptions not fully grounded in evidence to support their conclusions. Similarly, our study emphasizes how students’ evaluative criteria are shaped by prior knowledge and revised when confronted with anomalous evidence. In this way, the model complements componential approaches that break down CT into skills, dispositions, and background knowledge by making the mechanism of standard revision visible within a biology context (Hu & Bi, 2025).

The model also resonates with discipline-focused measurement and intervention work in science education, which shows that content mastery and context condition CT performance and its valid assessment (McMurray et al., 1991; Tiruneh et al., 2017). Our process account helps explain why domain-specific CT instruments in science (e.g., physics) must be tuned to disciplinary content: the quality of criteria in use mediates performance and transfer. By making this mediation explicit, the model offers an interpretive bridge between domain-general rubrics and domain-specific performance.

We read the findings through Kuhn’s cycle (normal science → anomaly → crisis → new framework) as an explanatory analogy, not as a strict identity claim. Following guidance from the HPS-in-science-education literature, we explicitly acknowledge two limits. First, we use Kuhn as a tool to understand how students’ evaluative criteria go through cycles of stability, disruption, and reorganization. This is not a direct application of Kuhn’s ideas on incommensurability or theory-ladenness to classroom thinking. Second, we recognize longstanding cautions against over-generalized relativism or simplistic one-to-one mappings in educational discourse; the value lies in clarifying the role of anomalies and how they precipitate revisions of criteria in learners’ reasoning, rather than in asserting that students undergo “mini-revolutions” in the full Kuhnian sense (Matthews, 2024).

Within these bounds, the analogy earns its keep in three ways. First, it highlights why routine success within established criteria (our “confirmation phase”) can coexist with blind spots to counter-instances—mirroring Kuhn’s description of puzzle-solving under a paradigm that sets legitimate problems and screens out anomalies. Second, it frames anomalies (e.g., genetic codon degeneracy) as productive cognitive disruptors that loosen prevailing evaluative criteria and invite their reconfiguration; this aligns with HPS-informed classroom accounts that leverage scientific controversies to surface presuppositions and prompt conceptual change (Liu et al., 2025b). Third, it legitimizes cyclical development—learners oscillate from confirmatory induction to deductive falsification and then to revised induction—echoing HPS arguments for integrating historical cases and structured conflicts to cultivate CT without sliding into relativism (Shan, 2020; Siegel, 2004).

To avoid over-extension of the analogy, we emphasize that our claims are empirically anchored in students’ written responses and classroom dialogues rather than speculative analogy alone. Our stance is consistent with scholarship urging careful use of Kuhn in education—foregrounding what Kuhn helps explain (e.g., how anomalies precipitate standard revision) while bracketing contentious theses (e.g., strong incommensurability) and explicitly excluding them from classroom practice (Matthews, 2024). In short, Kuhn functions here as a framing lens for a data-driven model, not as a doctrinal template.

Beyond theoretical alignment, the model carries practical and educational implications. By demonstrating how contradictory evidence destabilizes students’ prevailing criteria and forces revision, the findings suggest that instruction can deliberately use anomalies as catalysts for CT (Abrami et al., 2008; C. Park et al., 2023; J. Park, 2001). In biology classrooms, tasks such as genetic codon degeneracy or experimental outcomes that contradict naïve expectations can be staged to induce cognitive conflict and scaffold the questioning process. Rather than quickly correcting anomalies, they should be seen as valuable teaching tools that prompt students to reassess and refine their evaluative criteria. In this way, the model provides teachers with a process-level rationale for designing activities that promote iterative cycles of confirmation, falsification, and reconstruction. At the same time, the Kuhnian analogy gives educators a philosophical vocabulary for why such anomalies matter: they simulate the epistemic dynamics of science, underscoring that knowledge growth often comes from moments of disruption rather than accumulation alone (Mengistie & Worku, 2020). Together, the model and the analogy converge on a pedagogical message—teaching that makes anomalies visible and productively disruptive can help students develop the habits of questioning and standard revision that constitute CT.

Several limitations qualify these contributions. The data derive from students in one region of China, and although cross-regional sampling expanded the corpus, the findings are not yet sufficient to generalize broadly across cultural or curricular contexts. The focus on genetics tasks, while offering clear access to anomalies such as genetic codon degeneracy, raises the question of how robust the model is across other biology domains (e.g., ecology, evolution) or across different sciences (e.g., physics, chemistry). Moreover, the study was conducted through qualitative analytic procedures; while these yield process-rich accounts, complementary quantitative approaches—such as domain-specific CT assessments or mixed-method designs—would help test the generalizability and predictive utility of the model. Finally, the Kuhnian framing, though heuristically valuable, should not obscure alternative philosophical resources (e.g., Popper’s falsification, Lakatos’s research programs) that may illuminate different dimensions of CT development.

Future research can extend the present account in three directions. First, cross-disciplinary replications could test whether the core mechanism of questioning and standard revision is evident in other sciences and at different grade levels, thereby examining its generality while respecting disciplinary variation. Second, longitudinal studies could trace how students’ CT develops over time as they repeatedly encounter anomalies, providing evidence on durability and transfer. Third, broader philosophical dialogue could situate the model alongside alternative frameworks in HPS and contemporary cognitive science, helping delineate the distinct and overlapping insights each provides. Taken together, such work can refine the explanatory model, expand its evidentiary base, and integrate it into a wider landscape of CT research and pedagogy.

## 6. Conclusions

This study advanced a discipline-specific theoretical model of CT in senior secondary biology, developed through analytic integration of written responses and classroom dialogues on students’ engagement with contradictory evidence. At its core, the model identifies questioning as the process that drives the transformation of evaluative criteria, supported by complementary categories of reasoning, analytic moves, and the application of experiential knowledge. By tracing how learners shuttle between confirmation, falsification, and reconstruction, the study contributes an explanatory account of the mechanisms through which CT emerges in a concrete disciplinary context.

Through a heuristic dialogue with Kuhn’s account of scientific revolutions, the study further highlights the cyclical nature of CT development: anomalies can destabilize prevailing frameworks and trigger reorganizations of evaluative criteria, thereby catalyzing cognitive growth. This alignment underscores the pedagogical potential of deliberately designing learning environments that expose students to anomalies and cognitive conflicts, encouraging them to engage in questioning and refine their evaluative criteria.

In sum, this study situates CT within the authentic dynamics of disciplinary reasoning in biology by clarifying how anomalies trigger cycles of questioning and evaluative-criteria revision. By explicating these processes, the research offers a concise, discipline-specific framework that informs the design of instructional environments incorporating cognitive conflict, and provides a rationale for integrating history and philosophy of science (HPS)-informed strategies into biology classrooms.

## Figures and Tables

**Table 1:** Codebook for Critical Thinking Theory Construction.

Core Category	Major Category	Subcategory
Questioning: transforming multiple criteria	Evolving evaluation criteria	Judgment
		Criteria
		Questioning
		Comparison
		Reflection
	Various types of reasoning	Causal reasoning
		Abductive reasoning
		Deductive reasoning
		Inductive reasoning
	Analysis without judgment	Classification
		Explanation
		Identification
		Understanding
	The application of empirical knowledge to different standards	Hypotheses
		Knowledge-based classification
		Correct or incorrect interpretation
		Replication/recall
		Incorrect evidence
		Incomplete interpretation of information

## Data Availability

Requests for data can be sent to the corresponding author.

## Abbreviations

The following abbreviations are used in this manuscript:

CT	Critical thinking
HPS	History and philosophy of science
NOS	Nature of science

## Appendix A

**Table A1:** Representative Samples (19 Cases and Classroom Dialogue).

Sample ID	Group	Excerpt (Translated)	Analytic Labels (First-Cycle)	Link to Memo
05	A	“Gene mutation leads to protein change, and further causes phenotype change.”	Mutation → protein → trait causal chain	Memo F-1
07	B	“If mutation changes coding, the amino acids may be replaced, and then protein functions change.”	Evidence-based reasoning; conditional thinking	Memo F-2
17	B	“Not always. Some mutations have no effect.”	Exceptions; boundary conditions; uncertainty	Memo F-3
21	A	“Protein change can be caused by substitution or deletion, leading to disease.”	Mechanistic reasoning; linking mutation with disease	Memo F-4
24	A	“Sometimes mutation does not affect phenotype if it is silent.”	Awareness of silent mutation; evaluation of impact	Memo F-5
25	C	“Some mutations may change structure but not function, depending on amino acid properties.”	Structure–function reasoning; conditional evaluation	Memo F-6
26	B	“Mutation may cause hemoglobin abnormality, such as sickle-cell anemia.”	Using concrete examples; connecting to known cases	Memo F-7
33	C	“Environmental factors may trigger mutations, influencing biological traits.”	External causes; linking environment to mutation	Memo F-8
34	C	“DNA replication errors may cause mutations, influencing protein synthesis.”	Causal chain reasoning; process awareness	Memo F-9
44	D	“Mutations may directly affect traits like eye color.”	Simplified causal chain; phenotype-oriented	Memo F-10
47	A	“Coding sequence changes → amino acid substitution → protein changes → trait change.”	Multi-step reasoning; explicit chain	Memo F-11
48	A	“Not all mutations change amino acids; sometimes no effect occurs.”	Awareness of redundancy; exceptions	Memo F-12
60	B	“Mutations sometimes lead to beneficial results, sometimes harmful.”	Balanced view; dual outcomes	Memo F-13
63	C	“If mutation changes the coding, it may or may not affect phenotype.”	Conditional reasoning; uncertainty	Memo F-14
80	B	“Mutations can damage DNA sequences, leading to trait changes.”	Mutation → DNA damage → phenotype	Memo F-15
84	C	“Mutation in chlorophyll synthesis gene causes yellow leaves.”	Concrete biological example; evidence-based reasoning	Memo F-16
87	D	“Gene mutations lead to abnormal protein and phenotype changes.”	Causal chain; generalized reasoning	Memo F-17
91	D	“DNA sequence replacement or deletion causes phenotype changes.”	Specific mutation type; mechanistic awareness	Memo F-18
92	D	“Sometimes students ignore conflicting evidence and hold original view.”	Circumventing contradictions; coping strategy	Memo F-19
Classroom Dialogue	Participant 2	“Does the substitution occur at the same position?” “The positions differ.”	Conflict with prior hypothesis; questioning hypothesis	Memo F-20

## Appendix B

Memo Excerpts (Selective Coding Stage)

This appendix presents representative memo excerpts generated during selective coding. The memos illustrate early pattern recognition, category refinement, dialogue-based interpretation, and the identification of the core category.

Memo Excerpt 1 (Batch A, Case 05):

“Students tend to use a simple causal chain: mutation → protein → phenotype. This indicates early reliance on linear reasoning, which later becomes the foundation for building the category confirmation through supportive evidence.”

Memo Excerpt 2 (Batch B, Case 17):

“When faced with contradictions, some students explicitly note exceptions (‘not always’). This supports the sub-category of boundary conditions and links to the emerging core category of Questioning: Transforming Multiple Criteria.”

Memo Excerpt 3 (Batch C, Case 25):

“Responses considering amino acid properties reflect more advanced reasoning. This shows criteria-shifting behavior—students weigh structural versus functional impacts, aligning with the notion of multiple standards in judgment.”

Memo Excerpt 4 (Selective coding, core-category decision; across Batches A–C):

Across cases, moments of questioning consistently co-occur with shifts in evaluative criteria when students encounter conflicting evidence. Alternative candidate processes (e.g., comparison, explanation, or hypothesis generation) appear dependent on whether questioning is triggered. Therefore, questioning that transforms multiple criteria is treated as the core category because it organizes relationships among reasoning forms, analytic moves, and stability of criteria.

Memo Excerpt 5 (Batch D, Case 92—negative case):

“This student disregards conflicting evidence and circumvents the falsification stage. It demonstrates an alternative pathway, which was coded as circumvent contradiction. It serves as a boundary condition for the theoretical model.”

Memo Excerpt 6 (Classroom Dialogue, Participant 2)
  Excerpt (translated):
  Facilitator: “Does the substitution occur at the same position?”
  Student: “The positions differ.”
  Facilitator: “These conflicts with your hypothesis. Do you still hold on to your original idea?”
  Student: “Yes, I insist.”
  Analytic codes:
  Conflict with prior hypothesis; questioning hypothesis; resistance to revising
  Link to Memo:

This interaction demonstrates a key moment where the student encounters conflicting evidence but remains attached to their original hypothesis, resisting revision despite the evidence. This illustrates the student’s stubbornness in maintaining their initial reasoning, a potential boundary condition for the theoretical model. The student’s persistence in maintaining their hypothesis despite the contradiction suggests that some learners may hold on to their preconceived notions, even when faced with contrary evidence.

Memo Excerpt 7 (Classroom Dialogue, Participant 2)
  Excerpt (translated):
  Facilitator: “Different codons may correspond to the same polypeptide fragment.”
  Student: “Yes, I believe so.”
  Facilitator: “Does this diverge from your prior idea?”
  Student: “It does, but I still think it’s valid.”
  Analytic codes:
  Evidence-based reasoning; hypothesis revision; codon redundancy
  Link to Memo:

This exchange reveals the student’s recognition of codon redundancy, where different codons can encode the same amino acid. The student reflects on the implications for their previous hypothesis and is prompted to revise their thinking. This shows a shift towards more evidence-based reasoning as the student begins to adjust their previous understanding, aligning with the process of questioning and transforming multiple criteria. This revision suggests a change in the evaluative criteria used to assess genetic mutations and their effects on protein synthesis.

## Appendix C

**Table A2:** Saturation Log.

Batch	Cases Analyzed	New Properties?	New Relationships?	Memo Reference
A	5	Yes: added “Openness to Evidence”	Yes: linked reasoning chain to assumption-building	Memo A-1
B	5	Minimal: added “Boundary conditions”	Yes: strengthened link between contradiction and coping strategies	Memo B-2
C	5	No	No	Memo C-3 (saturation reached)

Conclusion: By Batch C, no new properties or inter-category relationships emerged. Therefore, theoretical saturation was judged to have been achieved.

## Appendix D

**Table A3:** External Validation (Batch D).

Case ID	Coding Outcome	Fit with Model?	Notes
44	Confirmed mutation → protein → phenotype chain	Yes	Confirms supportive evidence path
87	Mapped fully to “confirmation → falsification → new assumption” cycle	Yes	Confirms sequential cycle
91	Partial fit (skipped questioning stage)	Partial	Suggests some learners bypass criteria shift
92	Contradiction (ignored conflicting evidence)	Boundary Case	Used as negative case; supports inclusion of “circumvent contradiction” path

Summary: Most Batch D cases confirmed the model. A few provided partial or contradictory evidence, which were incorporated as boundary conditions, thereby enhancing the robustness of the theoretical model.

## References

[B1-behavsci-16-00296] Abrami P. C., Bernard R. M., Borokhovski E., Waddington D. I., Wade C. A., Persson T. (2015). Strategies for teaching students to think critically: A meta-analysis. Review of Educational Research.

[B2-behavsci-16-00296] Abrami P. C., Bernard R. M., Borokhovski E., Wade A., Surkes M. A., Tamim R., Zhang D. (2008). Instructional interventions affecting critical thinking skills and dispositions: A stage 1 meta-analysis. Review of Educational Research.

[B3-behavsci-16-00296] Altun E., Yildirim N. (2023). What does critical thinking mean? Examination of pre-service teachers’ cognitive structures and definitions for critical thinking. Thinking Skills and Creativity.

[B4-behavsci-16-00296] Arabatzis T., Kindi V., Vosniadou S. (2008). The problem of conceptual change in the philosophy and history of science. International handbook of research on conceptual change.

[B5-behavsci-16-00296] Bailin S. (2002). Critical thinking and science education. Science & Education.

[B6-behavsci-16-00296] Cardinot D., Moura C., Guerra A. (2023). Challenging the “Science from nowhere” perspective in the classroom. Science & Education.

[B7-behavsci-16-00296] Cargas S., Williams S., Rosenberg M. (2017). An approach to teaching critical thinking across disciplines using performance tasks with a common rubric. Thinking Skills and Creativity.

[B8-behavsci-16-00296] Cáceres M., Nussbaum M., Ortiz J. (2020). Integrating critical thinking into the classroom: A teacher’s perspective. Thinking Skills and Creativity.

[B9-behavsci-16-00296] Corbin J., Strauss A. (2015). Basics of qualitative research: Techniques and procedures for developing grounded theory.

[B10-behavsci-16-00296] Cuypers S. E. (2004). Critical thinking, autonomy and practical reason. Journal of Philosophy of Education.

[B11-behavsci-16-00296] Davies M. (2013). Critical thinking and the disciplines reconsidered. Higher Education Research & Development.

[B12-behavsci-16-00296] Ennis R. H., Davies M., Barnett R. (2015). Critical thinking: A streamlined conception. The Palgrave handbook of critical thinking in higher education.

[B13-behavsci-16-00296] Facione P. A. (1990). The Delphi report. Critical thinking: A statement of expert consensus for purposes of educational assessment and instruction.

[B14-behavsci-16-00296] García-Carmona A. (2025). Scientific thinking and critical thinking in science education. Science & Education.

[B15-behavsci-16-00296] Hart C., Da Costa C., D’Souza D., Kimpton A., Ljbusic J. (2021). Exploring higher education students’ critical thinking skills through content analysis. Thinking Skills and Creativity.

[B16-behavsci-16-00296] Hu X., Bi H. (2025). Exploring and validating the componential model of students’ scientific critical thinking in science education. Thinking Skills and Creativity.

[B17-behavsci-16-00296] Huang J., Sang G. (2023). Conceptualising critical thinking and its research in teacher education: A systematic review. Teachers and Teaching.

[B18-behavsci-16-00296] Kuhn T. S. (1962). The structure of scientific revolutions.

[B19-behavsci-16-00296] Lead States N. G. S. S. (2013). Next generation science standards: For states, by states.

[B20-behavsci-16-00296] Li X., Liu J. (2021). Mapping the taxonomy of critical thinking ability in EFL. Thinking Skills and Creativity.

[B21-behavsci-16-00296] Liu W., Li X., Li G. (2025a). The contributions of philosophy of science in science education research: A literature review. Science & Education.

[B22-behavsci-16-00296] Liu W., Liu B., Chen C., Han Y., Li G. (2025b). Analyzing students’ critical thinking processes based on falsification heuristic experiment. Thinking Skills and Creativity.

[B23-behavsci-16-00296] Loving C. C., Cobern W. W. (2000). Invoking Thomas Kuhn: What citation analysis reveals about science education. Science & Education.

[B24-behavsci-16-00296] Matthews M. R. (2004). Thomas Kuhn’s impact on science education: What lessons can be learned?. Science Education.

[B25-behavsci-16-00296] Matthews M. R. (2024). Thomas Kuhn and science education. Science & Education.

[B26-behavsci-16-00296] McMurray M. A., Beisenherz P., Thompson B. (1991). Reliability and concurrent validity of a measure of critical thinking skills in biology. Journal of Research in Science Teaching.

[B27-behavsci-16-00296] McPeck J. E. (1990). Teaching critical thinking: Dialogue and dialectic.

[B28-behavsci-16-00296] Mengistie S., Worku M. (2020). Lessons educators could learn from Thomas Kuhn’s the structure of scientific revolutions. International Journal of Education & Management Studies.

[B29-behavsci-16-00296] Ministry of Education of the People’s Republic of China (2018). Biology curriculum standards for senior high school *[普通高中生物课程标准 (2017版)]*.

[B30-behavsci-16-00296] Mkimbili S. T. (2024). Do biology syllabi provide opportunities for secondary school students to engage with critical thinking skills?. Journal of Biological Education.

[B31-behavsci-16-00296] Moeiniasl H., Taylor L., deBraga M., Manchanda T., Huggon W., Graham J. (2022). Assessing the critical thinking skills of English language learners in a first year psychology course. Thinking Skills and Creativity.

[B32-behavsci-16-00296] Moore T. (2004). The critical thinking debate: How general are general thinking skills?. Higher Education Research & Development.

[B33-behavsci-16-00296] Moore T. (2013). Critical thinking: Seven definitions in search of a concept. Studies in Higher Education.

[B34-behavsci-16-00296] Moore T. J. (2011). Critical thinking and disciplinary thinking: A continuing debate. Higher Education Research & Development.

[B35-behavsci-16-00296] Moura C. B., Alsop S., Camel T., Guerra A. (2023). Science education in a world in crisis: Contributions from the South to a defense of a cultural–historical approach in science teaching. Cultural Studies of Science Education.

[B36-behavsci-16-00296] Mulnix J. W. (2012). Thinking critically about critical thinking. Educational Philosophy and Theory.

[B37-behavsci-16-00296] Niaz M. (2010). Science curriculum and teacher education: The role of presuppositions, contradictions, controversies and speculations vs. Kuhn’s ‘normal science’. Teaching and Teacher Education.

[B38-behavsci-16-00296] Oh J. Y. (2017). Suggesting a NOS map for nature of science for science education instruction. Eurasia Journal of Mathematics, Science and Technology Education.

[B39-behavsci-16-00296] Park C., Mun S., Hong H. G. (2023). High school students’ evolving alternative conception related to the volume of gas: A Lakatosian perspective. Journal of Research in Science Teaching.

[B40-behavsci-16-00296] Park J. (2001). Analysis of students’ processes of confirmation and falsification of their prior ideas about electrostatics. International Journal of Science Education.

[B41-behavsci-16-00296] Paul R., Elder L. (2013). Critical thinking: Tools for taking charge of your professional and personal life.

[B42-behavsci-16-00296] Paulsen V. H., Kolstø S. D. (2022). Students’ reasoning when faced with test items of challenging aspects of critical thinking. Thinking Skills and Creativity.

[B43-behavsci-16-00296] Putra P. D. A., Sulaeman N. F., Supeno, Wahyuni S. (2023). Exploring students’ critical thinking skills using the engineering design process in a physics classroom. The Asia-Pacific Education Researcher.

[B44-behavsci-16-00296] Renaud R. D., Murray H. G. (2008). A comparison of a subject-specific and a general measure of critical thinking. Thinking Skills and Creativity.

[B45-behavsci-16-00296] Schindler S. (2013). The Kuhnian mode of HPS. Synthese.

[B46-behavsci-16-00296] Shan Y. (2020). Kuhn’s “wrong turning” and legacy today. Synthese.

[B47-behavsci-16-00296] Shi W.-Z. (2015). Utilizing history and philosophy of science (HPS) to teach physics: The case of electromagnetic theory. Eurasia Journal of Mathematics, Science and Technology Education.

[B48-behavsci-16-00296] Shi X. (2025). Improving the argumentation abilities of high school students in China via the Toulmin argumentation pattern, Popper’s falsificationism, and the game of Eleusis. Science & Education.

[B49-behavsci-16-00296] Siegel H. (2004). The bearing of philosophy of science on science education, and vice versa: The case of constructivism. Studies in History and Philosophy of Science Part A.

[B50-behavsci-16-00296] Sparks J. R., Rapp D. N. (2011). Readers’ reliance on source credibility in the service of comprehension. Journal of Experimental Psychology: Learning, Memory, and Cognition.

[B51-behavsci-16-00296] Tian L., Gai L., Huo Y., Wang J., Dong H., Zhang L. (2025). Theoretical framework and assessment questionnaire of critical thinking for Chinese middle school students in science classrooms. Science & Education.

[B52-behavsci-16-00296] Tiruneh D. T., De Cock M., Weldeslassie A. G., Elen J., Janssen R. (2017). Measuring critical thinking in physics: Development and validation of a critical thinking test in electricity and magnetism. International Journal of Science and Mathematics Education.

[B53-behavsci-16-00296] Willingham D. T. (2008). Critical thinking: Why is it so hard to teach?. Arts Education Policy Review.

[B54-behavsci-16-00296] Wray K. B. (2011). Kuhn and the discovery of paradigms. Philosophy of the Social Sciences.

[B55-behavsci-16-00296] Yan Z. (2021). English as a foreign language teachers’ critical thinking ability and L2 students’ classroom engagement [Mini Review]. Frontiers in Psychology.

